# Kinins’ Contribution to Postoperative Pain in an Experimental Animal Model and Its Implications

**DOI:** 10.3390/brainsci13060941

**Published:** 2023-06-12

**Authors:** Indiara Brusco, Cássia Regina Silva, Juliano Ferreira, Sara Marchesan Oliveira

**Affiliations:** 1 Graduate Program in Biological Sciences: Biochemical Toxicology, Center of Natural and Exact Sciences, Federal University of Santa Maria, Camobi, Santa Maria 97105-900, RS, Brazil; 2 Graduate Program in Environmental Sciences, Universidade Comunitária da Região de Chapecó, Chapecó 89809-000, SC, Brazil; 3 Graduate Program in Genetics and Biochemistry, Institute of Biotechnology, Federal University of Uberlândia, Uberlândia 38401-136, MG, Brazil; cassia.regina@ufu.br; 4 Graduated Program in Pharmacology, Pharmacology Department, Federal University of Santa Catarina, Florianopolis 88040-900, SC, Brazil; ferreiraj99@gmail.com; 5 Department of Biochemistry and Molecular Biology, Federal University of Santa Maria, Camobi, Santa Maria 97105-900, RS, Brazil

**Keywords:** analgesics, allodynia, antinociception, kinins, surgical pain

## Abstract

Postoperative pain causes discomfort and disability, besides high medical costs. The search for better treatments for this pain is essential to improve recovery and reduce morbidity and risk of chronic postoperative pain. Kinins and their receptors contribute to different painful conditions and are among the main painful inflammatory mediators. We investigated the kinin’s role in a postoperative pain model in mice and reviewed data associating kinins with this painful condition. The postoperative pain model was induced by an incision in the mice’s paw’s skin and fascia with the underlying muscle’s elevation. Kinin levels were evaluated by enzyme immunoassays in sham or operated animals. Kinin’s role in surgical procedure-associated mechanical allodynia was investigated using systemic or local administration of antagonists of the kinin B_1_ receptor (DALBk or SSR240612) or B_2_ receptor (Icatibant or FR173657) and a kallikrein inhibitor (aprotinin). Kinin levels increased in mice’s serum and plantar tissue after the surgical procedure. All kinin B_1_ or B_2_ receptor antagonists and aprotinin reduced incision-induced mechanical allodynia. Although controversial, kinins contribute mainly to the initial phase of postoperative pain. The kallikrein–kinin system can be targeted to relieve this pain, but more investigations are necessary, especially associations with other pharmacologic targets.

## 1. Introduction

Most surgical patients experience acute postoperative pain related to injury, which is reported as moderate, severe, or extreme by approximately 75% of them [1]. Evidence suggests that less than 50% of patients undergoing a surgical procedure present adequate postoperative pain relief, compromising their life quality and functional recovery. Importantly, pain is a frequent cause of readmission and seeking medical care after hospital discharge [1,2]. Moreover, in approximately 10% of patients, uncontrolled acute postoperative pain can progress beyond the usual tissue healing time to a persistent pain condition in the incision site or related areas, which persists for three months or more [2,3]. The persistent postoperative pain symptoms more frequently reported include allodynia (pain in response to normally non-painful stimuli), hyperalgesia (increased pain in response to painful stimuli), and dysesthesia (an unpleasant, abnormal sensitivity to touch) [3].

Globally, over 320 million surgical procedures have been estimated by the year, representing a vast potential for developing chronic postoperative pain [3,4]. Remarkably, acute postoperative pain management influences pain chronicity since adequate analgesic treatment during hospitalization results in better pain control and functional recovery and decreases the risk of complications [2,5,6]. Unfortunately, the concept of preventive analgesia to reduce central sensitization and persistent pain has been disappointing [6]. Moreover, over the past decade, there has been a reliance on opioids to treat postoperative pain contributing to a rising epidemic of opioid misuse, abuse, and overdose-related deaths [3]. Despite advances, optimizing acute postoperative pain management to reduce chronic postoperative pain remains a significant challenge since this pain is a health problem due to morbidity, hospital expenses, and psychological distress [3,6,7]. Studies with local and regional anesthetics, non-steroidal anti-inflammatory drugs, glutamatergic antagonists, and antiepileptic and antidepressant drugs have been disappointing and have shown that most pharmacological interventions are ineffective in preventing the development of persistent postoperative pain [3]. Therefore, there is a need to integrate clinical epidemiology and basic science research to advance in managing postoperative pain and preventing persistent postoperative pain [1,2].

Analgesic strategies that target neuroinflammatory responses are suggested as promising to improve postoperative pain treatment [6] since surgical injury leads to a complex cascade of events mediated by nociceptive neurons and inflammatory and immunological responses, which contribute to the onset of pain [2,3,6]. Kinins and inflammatory and nociceptive mediators are among the potential peripheral mechanisms suggested for postoperative pain development [8,9,10]. In response to inflammation or tissue damage, kinins (bradykinin and kallidin) are formed and activate kinin B_2_ receptors, while their active metabolites (des-Arg-kinins) activate kinin B_1_ receptors [9,10,11]. The activation of these receptors triggers a cascade of intracellular events involved in different types of pain [10], such as the inflammatory pain associated with arthritis [12,13], gout [14], fracture [15], and ischemia [16], among others. Notably, kinin levels and B_1_ and B_2_ receptor mRNA expression increased in the microdialysate and mucosal biopsies after oral surgical procedures in humans [17,18]. Moreover, some studies with animal models have tried to elucidate the role of kinins in postoperative pain [19,20,21], but these data remain inconclusive. In this way, we investigated the involvement of kinins in a postoperative pain model in mice besides discussing existing data on the relation of kinins with postoperative pain.

## 2. Materials and Methods

### 2.1. Drugs and Reagents

Icatibant (peptide kinin B_2_ receptor antagonist) and des-Arg^9^-[Leu^8^]-bradykinin (DALBk; peptide kinin B_1_ receptor antagonist) were obtained from Sigma Chemical Company (St. Louis, MO, USA). FR173657 (non-peptide kinin B_2_ receptor antagonist) and SSR240612 (non-peptide kinin B_1_ receptor antagonist) were obtained from Sanofi-Aventis (Germany). Aprotinin was acquired from Sigma-Aldrich Chemical Company (St. Louis, MO, USA). Kinin receptor antagonists and aprotinin were prepared in a saline solution (0.9% NaCl), which was used as the vehicle in control groups. The enzyme immunoassay kit for bradykinin was obtained from Peninsula Laboratories International, Inc. (San Carlos, CA, USA). The doses of the drugs used in this study were based on pilot experiments or previous studies. They were administered in a volume of 10 mL/kg (systemic administration) or 20 μL/paw (intraplantar injection) [22,23].

### 2.2. Animals

The experiments were conducted using 208 adult male Swiss mice (25–30 g; 6–7 weeks of age) produced and provided by the Federal University of Santa Maria. The animals were maintained in a temperature-controlled room (22 ± 1 °C) under a 12 h light/12 h dark cycle with free access to food and water. Experimental protocols were performed with the approval of the Institutional Animal Care and Use Committee of the Federal University of Santa Maria (process #00107). Experimental protocols were conducted according to the guidelines for investigation of experimental pain in conscious animals [24], national and international legislation (guidelines of the Brazilian Council of Animal Experimentation Control—CONCEA—and the U.S. Public Health Service’s Policy on Humane Care and Use of Laboratory Animals—PHS policy), and Animal Research: Reporting in vivo Experiments (ARRIVE) guidelines [25,26]. The number of animals and the intensities of noxious stimuli used were the minimum necessary to demonstrate the consistent effects of the treatments. The group size for each experiment was based on studies with protocols similar to ours [22,27,28,29], which were confirmed by power calculations (G*Power version 3.1.9.7).

### 2.3. Postoperative Pain Model

The postoperative pain model in mice was performed as previously described [27,28,29]. Firstly, mice were anesthetized with 2% isoflurane via a nose cone. After anesthesia and anti-septic preparation of the right hind paw with 10% povidone-iodine solution (PVPI), a 5 mm longitudinal incision was made through the skin and fascia of the plantar foot with a number 11 blade. The incision began 2 mm from the proximal edge of the heel and extended toward the toes. The underlying muscle was elevated with curved forceps, leaving the muscle origin and insertion intact, and the skin was sutured with a single mattress suture of 6.0 nylon.

### 2.4. Bradykinin-Related Peptides Levels

We verified the bradykinin-related peptide levels after the sham or surgical procedure in mice without treatment. For this, mice submitted to surgical procedure or sham (mice just anesthetized but not operated) were euthanized 1 h after the procedure. The plantar tissue was collected and immediately frozen in liquid nitrogen and homogenized in phosphate-buffered saline containing a cocktail of kininase inhibitors. Then, the samples were lyophilized and resuspended in the assay buffer. The blood collected by cardiac puncture was centrifuged to obtain serum which also received the cocktail containing kininase inhibitors. The bradykinin-related peptide levels were measured by enzyme immunoassays using a high-sensitivity kit for bradykinin. For reaction, 50 µL of the sample, 25 µL of antiserum, and 25 µL of Bt-tracer were incubated for 2 h, followed by the administration of 100 µL of streptavidin and chromogenic substrate as per the manufacturer’s instructions. The reaction was terminated with 100 µL of 2N HCl, and absorbances were determined at 450 nm. The results were expressed as bradykinin-related peptide levels in ng/mg protein to serum and ng/mL/g tissue to the paw tissue [22,23].

### 2.5. Mechanical Allodynia Assessment

To evaluate the mechanical paw withdrawal threshold (PWT) of mice, they were placed individually in clear plexiglass boxes (7 × 9 × 11 cm) on elevated wire mesh platforms to allow access to the ventral surface of the hind paws. They remained in the box for approximately 1.5 h for behavioral accommodation before procedures. The mechanical PWT was determined before (basal measure) and after the surgical procedure with flexible nylon von Frey filaments using the Up-and-Down method [27,28,29,30]. A decrease in the mechanical PWT when compared with the same paw before surgery (basal value) was considered mechanical allodynia.

### 2.6. Treatments

The animals received systemic administration (subcutaneously via, s.c.) of the peptide selective B_1_ receptor antagonist DALBk (10, 30, and 100 nmol/kg, s.c., *n* = 6–9 animals by group) or B_2_ receptor antagonist Icatibant (300, 600, and 1000 nmol/kg, s.c., *n* = 8) or vehicle (saline 10 mL/kg, s.c., *n* = 8–9) 0.5 h before the surgical procedure. Likewise, a non-peptide selective B_1_ receptor antagonist (SSR240612, 100, 300, and 1000 nmol/kg, s.c., *n* = 5–8), a non-peptide selective B_2_ receptor antagonist (FR173657, 30, 100, and 300 nmol/kg, s.c., *n* = 6–8), or vehicle (Saline, 10 mL/kg, s.c., *n* = 8–9) was also administered 0.5 h before surgery. Moreover, other groups of animals received the local treatment (intraplantar via, i.pl.) of DALBk (0.1, 0.3, and 1 nmol/paw, i.pl., *n* = 7–8), Icatibant (1, 3, and 10 nmol/paw, i.pl., *n* = 7–8), or vehicle (Saline, 20 µL/paw, i.pl., *n* = 8) 10 min before the surgical procedure. The aprotinin (100 µg/paw, i.pl., *n* = 8), an inhibitor of serine proteases such as kallikrein, also was administered intraplantarly (10 min) before surgery. The dosages and timing of the drugs were obtained from pilot experiments or previous studies [22,23]. The mechanical PWT was assessed 1, 2, and 4 h after the surgical procedure to determine treatment effects.

### 2.7. Statistical Analysis

Statistical analyses were carried out using Graph Pad Prism 8.0 software (Graph Pad, San Diego, CA, USA). Results were expressed as the mean + standard error of the mean (SEM). The significance of differences between groups was evaluated with Student’s *t*-test or two-way (time and treatment as factors; F values indicate the interaction between these factors) analysis of variance (ANOVA) followed by Tukey’s post hoc test or Bonferroni’s post hoc test. To meet the parametric assumptions, the data on the mechanical threshold were log-transformed before analyses. The percentages of maximum effect were calculated for the maximal developed responses compared to baseline values or the control group. *p*-values less than 0.05 (*p* < 0.05) were considered statistically significant.

## 3. Results

### 3.1. The Surgical Procedure Increases Bradykinin-Related Peptide Levels

**Figure 1:**
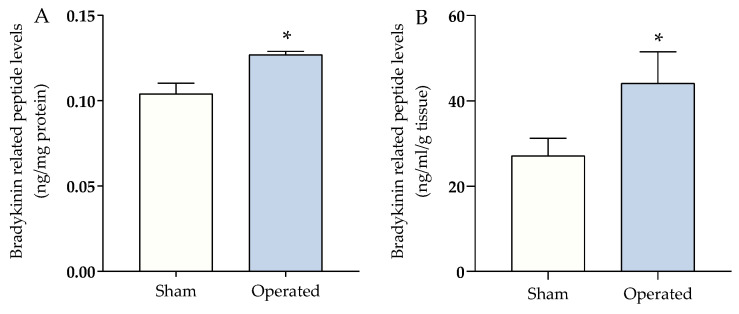
Bradykinin-related peptide levels in the serum (**A**) and plantar tissue (**B**) of mice submitted to surgical procedure. Data were expressed as the mean + SEM (*n* = 4–5/group to A and *n* = 6/group to B) and analyzed by Student’s *t*-test. * *p* < 0.05 compared with the respective sham (non-operated) group.

Bradykinin-related peptide levels increased in the serum (22 ± 1.8%) (Figure 1A) and paw tissue (91 ± 41%) (Figure 1B) of mice submitted to the procedure (operated) compared to mice sham (mice just anaesthetized but not operated) at 1 h after surgical procedure.

### 3.2. Kinin Receptor Antagonists and a Kallikrein Inhibitor Alleviate the Surgical Procedure-Induced Mechanical Allodynia in Mice

**Figure 2:**
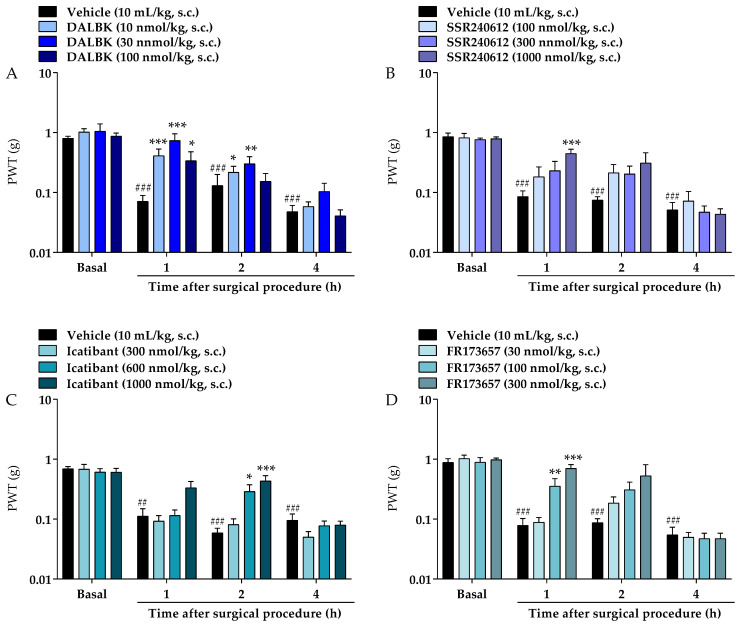
Effects of systemic pre-treatment (0.5 h before surgery) with DALBk (10, 30, or 100 nmol/kg, s.c.) (**A**), SSR240612 (100, 300, or 1000 nmol/kg, s.c.) (**B**), Icatibant (300, 600, or 1000 nmol/kg, s.c.) (**C**) or FR173657 (30, 100, or 300 nmol/kg, s.c.) (**D**) on the mechanical allodynia caused by surgical procedure in mice. Data were expressed as the mean + SEM (*n* = 5–9/group) and analyzed by two-way ANOVA followed by Tukey’s post hoc test. ^##^ *p* < 0.01; ^###^ *p* < 0.001 compared with basal mechanical PWT; * *p* < 0.05, ** *p* < 0.01, *** *p* < 0.001 compared with respective control group. PWT: paw withdrawal threshold.

The systemic pre-treatment with DALBk (10, 30, and 100 nmol/kg, s.c.) reduced the mechanical allodynia of mice caused by surgical procedure in all tested doses compared to the control group (F (9, 75) = 2.242; *p* = 0.0281; Figure 2A). This effect occurred from 1 to 2 h in the doses of 10 and 30 mg/kg and at 1 h in the dose of 100 mg/kg after the surgical procedure with maximum inhibition (I_max_) of 91 ± 30% in the dose of 30 nmol/kg at 1 h. Similar to DALBk, the non-peptide selective B_1_ receptor antagonist SSR240612 administered subcutaneously also reduced the mechanical allodynia only in the higher dose of 1000 nmol/kg (I_max_ of 47 ± 9.8%) compared to the control group (F (9, 75) = 1.846; *p* = 0.0738; Figure 2B). This effect occurred only in 1 h after the surgical procedure.

The systemic pre-treatment with Icatibant reduced the mechanical allodynia of mice caused by surgical procedure compared to the control group. This effect was observed in the doses of 600 and 1000 nmol/kg at 2 h after surgery with an I_max_ of 59 ± 16% in the highest dose (F (9, 84) = 2.589; *p* = 0.0110; Figure 2C). Similar to Icatibant, the non-peptide selective B_2_ receptor antagonist FR173657 reduced the mechanical allodynia in the doses of 100 and 300 nmol/kg at 1 h after surgical procedure with I_max_ of 77 ± 13% in the highest dose (F (9, 78) = 2.973; *p* = 0.0043; Figure 2D).

**Figure 3:**
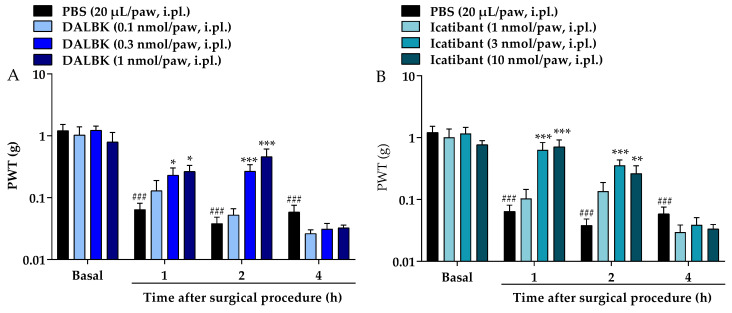
Effects of local pre-treatment (10 min before surgery) with DALBk (0.1, 0.3, or 1.00 nmol/paw, i.pl.) (**A**) or Icatibant (1, 3, or 10 nmol/paw, i.pl.) (**B**) on the mechanical allodynia caused by surgical procedure in mice. Data were expressed as the mean + SEM (*n* = 7–8/group) and analyzed by two-way ANOVA followed by Tukey’s post hoc test. ^###^ *p* < 0.001 compared with basal mechanical PWT; * *p* < 0.05, ** *p* < 0.01, *** *p* < 0.001 compared with respective control group. PWT: paw withdrawal threshold.

The local pre-treatment with kinin B_1_ and B_2_ receptor antagonists, administered 10 min before the surgical procedure, also attenuated the mechanical allodynia of mice. DALBk reduced the mechanical allodynia in doses of 0.3 and 1 nmol/paw compared to the control group. This effect occurred from 1 to 2 h after the surgical procedure with I_max_ of 35 ± 13% to the dose of 1 nmol/paw at 2 h (F (9, 78) = 4.481; *p* < 0.0001; Figure 3A). Icatibant also reduced the mechanical allodynia of mice in the doses of 3 and 10 nmol/paw, compared control group. This effect occurred from 1 to 2 h after the surgical procedure with an I_max_ of 56 ± 18% at 1 h after surgery in the dose of 10 nmol/paw (F (9, 78) = 6.431; *p* < 0.0001; Figure 3B).

**Figure 4:**
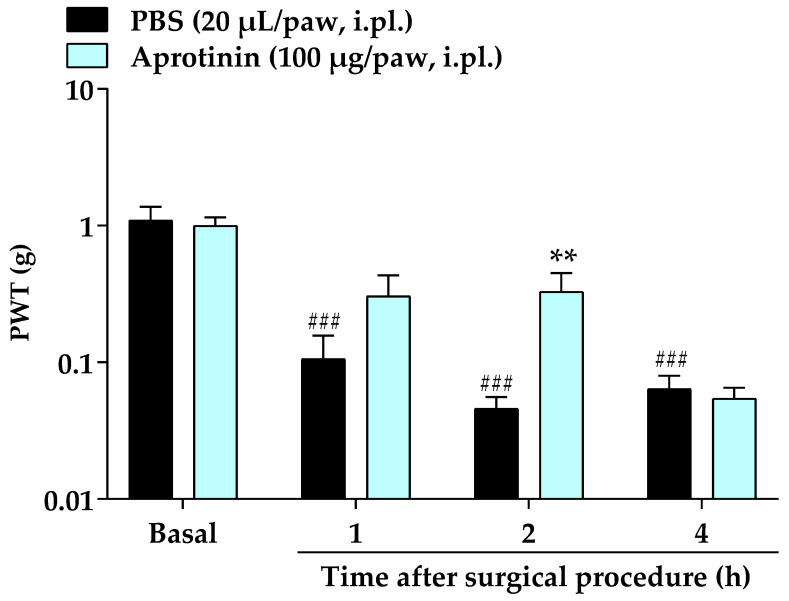
Effects of local pre-treatment (10 min before surgery) with aprotinin (100 µg/paw, i.pl.) on the mechanical allodynia caused by surgical procedure in mice. Data were expressed as the mean + SEM (*n* = 8/group) and analyzed by two-way ANOVA followed by Bonferroni’s post hoc test. ^###^ *p* < 0.001 compared with basal mechanical PWT; ** *p* < 0.01 compared with vehicle group. PWT: paw withdrawal threshold.

The local pre-treatment with aprotinin (100 µg/paw, i.pl.), an inhibitor of serine proteases such as kallikrein, administered 10 min before the surgical procedure, also attenuated the mechanical allodynia of mice at 2 h after its administration with inhibition of 33 ± 11% (F (3, 42) = 3.271; *p* = 0.0304; Figure 4).

## 4. Discussion

Postoperative pain is a common type of acute inflammatory pain faced by patients after surgical procedures, which is inadequately treated in more than half of cases [1,2,31]. Poorly managed postoperative pain can potentially acquire chronic characteristics of a complex, multifaceted pain syndrome [2,3]. Chronic postoperative pain has a high chance of developing due to millions of surgeries occurring annually worldwide [3,4]. It affects physical function and is accompanied by sleep disorders, anxiety, and depression, which compromises the patients’ quality of life [2,3]. Precocious postoperative pain management is a prerequisite for improving recovery and decreasing the risk of future complications [6]. However, despite an increased understanding of acute postoperative pain pathophysiology, developing new analgesics to adequately alleviate postoperative pain still needs to be improved [3,6,7].

Kinins play a role important in tissue inflammation and are one of the most potent pain-producing mediators in inflammatory conditions, being, therefore, suggested as involved in the pathophysiology of postoperative pain [2,9]. Although inconclusive, some studies investigated the kinin involvement in postoperative pain in experimental animals [19,20,21], which were discussed here (Table 1). Moreover, we showed that surgical procedure increased the bradykinin-related peptide levels in the paw tissue and serum of operated mice while the systemic or local treatment with kinin B_1_ and B_2_ receptor antagonists and a kallikrein inhibitor reduced mice’s mechanical allodynia induced by surgical procedure. These results suggest that the kallikrein–kinins system is an attractive pharmacological target to alleviate postoperative pain.

The postoperative pain caused by tissue trauma or injury and subsequent inflammatory response is categorized as inflammatory pain, whose immediate objective is to deal with the consequences of damage [2,31,32]. Typically, inflammatory pain is present only while the inflammation persists [32]. However, persistent noxious signaling in the periphery and maladaptive neuroplastic changes in the central nervous system contribute to chronic postoperative pain that can increase in intensity and last from days to months after the surgical procedure [2,3,32]. Although this pain often presents neuropathic pain mechanisms, chronic pain after surgery should be classified as postoperative pain [33,34].

In an individual operated upon, painful symptoms are localized to the surgical field or area of injury, projected to the innervation territory of a nerve situated in this area, or referred to a dermatome depending on the type of surgery and injury [3,33]. Here, we observed that a plantar incision reduced mice’s mechanical paw withdrawal threshold, characterizing the development of mechanical allodynia in the operated area. Notably, inflammatory pain is characterized most prominently by allodynia, a decurrent of reduced touch tenderness in surgical wounds or the next areas [32]. In this sense, pain evoked by everyday environmental stimuli such as gentle touch and similar pressure from clothing contact [35] can significantly affect patients’ quality of life. The plantar incision model has been used since the 90s to identify the mechanisms inherent in postoperative pain, where the transection of the skin and fascia and muscle retraction are compared to the tissue trauma and pain symptoms of patients undergoing surgery [7]. After the plantar incision procedure, nociceptors are sensitized, contributing to the onset of pain symptoms such as mechanical hypersensitivity [7,36,37,38].

Since surgical procedures active cascade events involving inflammatory and immunological responses and also activate nociceptive neurons to trigger the pain [2,3,6], analgesics targeting neuroinflammatory responses are fundamental to reducing persistent postoperative pain [3,6]. Surgical injury initiates an acute inflammatory reaction associated with the release of pro-inflammatory neuropeptides from peptidergic C-fibers in the area of injury [2]. These neuropeptides contribute to neuroplasticity after inflammatory injury once non-nociceptive fibers that normally transduce touch and proprioception acquire nociceptive characteristics, a peripheral mechanism behind allodynia or touch-induced pain [2,39]. Besides neuropeptides, pro-inflammatory mediators released from damaged cells in the injury local recruit more inflammatory cells that release cytokines and nerve growth factors at the injury site, enhancing the inflammatory process and, thus, the painful sensibility [2]. In this context, kinins are among these mediators that sustain tissue inflammation and can sensitize nociceptors to different stimuli, such as mechanical stimuli [2,9]. They are suggested as potential peripheral mechanisms associated with pain symptoms after surgical injury by activating kinin B_1_ and B_2_ receptors [2,9,10].

Peripheric and central nociceptive neurons and non-neuronal cells express kinin B_1_ and B_2_ receptors, whose activation triggers cellular excitation, explaining the ability of kinins to contribute to different pain conditions [10,11,14,40,41,42,43,44]. Notably, kinin B_1_ and B_2_ receptor mRNA expression was up-regulated in mucosal biopsies three hours after oral surgery in humans, possibly in response to kinin activation and cytokine and chemokine secretion [18]. Increased kinin levels also were found in microdialysate samples and mucosal biopsies obtained from these patients [17,18]. Corroborating these clinical data, we found increased bradykinin-related peptide levels in mice’s serum and plantar tissue submitted to the postoperative pain model induced by plantar incision. This increase is also found in mice’s inflammatory, neuropathic, and nociplastic pain models [14,22,23].

Since the bradykinin-related peptide levels increased after the surgical procedure, we considered it essential to investigate the effect of kinin B_1_ and B_2_ receptor antagonists in this postoperative pain model. Not surprisingly, systemic or local pre-treatment with kinin B_1_ and B_2_ receptor antagonists reduced mechanical allodynia induced by plantar incision. DALBk, a kinin B_1_ receptor antagonist, was more effective in reducing mechanical allodynia when administered systemically than locally. On the other hand, Icatibant, a kinin B_2_ receptor antagonist, presented similar efficacy in reducing mechanical allodynia by both systemic and local routes. Aprotinin, an inhibitor of serine proteases such as kallikrein, plasma and tissue enzymes that produce bradykinin and kallidin from high and low molecular weight kininogens, respectively [10,11], also reduced mechanical allodynia when administered intraplantar via in this pain model. Although aprotinin had a lower efficacy of inhibition of mechanical allodynia than the kinin receptor antagonists, the data suggest that the blockade of kinin receptors or reduced production of bradykinin and kallidin is involved in the antinociceptive effect observed in this postoperative pain model. In addition to systemic treatment, specific analgesic techniques, such as regional analgesia, were suggested to improve patient outcomes [45], and the kallikrein–kinin system may be an effective target for these two treatment alternatives. Together, our results corroborate Hamza and colleagues [18], who suggested that tissue injury associated with oral surgery in humans results in kinins production, which activates B_1_ and B_2_ receptors on nerve endings and, possibly, epithelial cells and gingival fibroblasts, contributing to surgical procedure-induced inflammatory pain.

The role of kinin receptors in postoperative pain has already been explored, and some data seem contradictory (Table 1). Leonard et al. [19] observed that DALBk or HOE140 (Icatibant) (0.1–3 mg/kg) intravenously administered 2 h or 2 days after incision did not alter mechanical or heat hypersensitivity caused by the plantar incision model in rats. This protocol measured behavioral tests from 0.5 to 2.5 h after treatments. Pre-treatment with the higher doses of DALBk or HOE140 (3 mg/kg), 1 h before incision, also did not prevent the mechanical and thermal hypersensitivity induced by the incision. In the pre-treatment protocol, the behavioral tests were evaluated 2 h after incision on the day of the operation, i.e., 3 h after antagonist administrations. One day after the surgical procedure, drugs were re-dosed, and 1 h later, the rats were tested for nociceptive behaviors, confirming the lack of antinociceptive effect of kinin antagonists. The authors concluded that kinin receptor antagonists have no antinociceptive effect on the postoperative pain model [19].

One year later, Muratani and colleagues [20] found that kinin B_1_ receptor antagonist [des-Arg^10^]-HOE140 (0.2 mg/kg) did not reduce mechanical hypersensitivity when administered subcutaneously 30 min before plantar incision in mice. Conversely, the kinin B_2_ receptor antagonist, HOE140 (0.02 and 0.2 mg/kg), reduced the mechanical hypersensitivity dose-dependent from 2 h to 3 days after the operation when administered subcutaneously 30 min before plantar incision. However, HOE140, administered 5 min after the surgical procedure, reduced mechanical hypersensitivity only at the highest dose tested 2 h after the operation. All behavioral tests were analyzed at 2 h after incision or once daily from day 1 to day 5 after the operation. Moreover, kinin B_1_ and B_2_ receptor mRNA expression did not change in the plantar tissue ipsilateral to the incision or spinal cord 24 h after the operation, and HOE140 (30 min pre-treatment) also did not affect this expression. The study suggested that kinin B_2_ receptor blockade may be effective in preventive analgesia to attenuate postoperative pain [20].

Next, Füredi et al. [21] showed that kinin B_1_ and B_2_ receptor antagonists, [des-Arg^10^]-HOE140 and HOE140, respectively, intraplantarly injected (10 µM) 18 h after surgical procedure alleviated the heat hypersensitivity induced by plantar incision in rats from 10 to 40 min after treatment. In this study, lipoxygenase or nitric oxide synthase inhibitors and P2X purinoceptor or transient receptor potential vanilloid 1 (TRPV1) receptor antagonists also reduced the heat hypersensitivity induced by surgical procedure [21]. Notably, a TRPV1 modulation mechanism via protein kinase-C (PKC) is suggested for the bradykinin-induced excitation of nociceptors and their sensitization to heat [46,47]. Thus, activating B_1_ and B_2_ receptors by kinins can lead the PKC activation with consequent TRPV1 phosphorylation, contributing to surgical procedure-evoked heat hyperalgesia [21].

The discrepancies found in studies investigating the role of kinins in postoperative pain models may be associated with differences in the doses of antagonists, via time of administration (pre- or post-treatment), time of evaluation of nociceptive behaviors after treatments, methodologies for behavioral experiments, and the plantar incision model (with or without muscle elevation or incision) (Table 1). Preventive and local treatment, i.e., peripheral actions and nociceptive behaviors evaluated within the first 2 h of the antagonist administration, seem to be more effective. Moreover, kinin receptor antagonists seem to partially alleviate some nociceptive behaviors, which can be advantageous in postoperative pain treatment. Surgical wound healing could be impaired if postoperative pain is wholly reduced and patients are excessively active after surgery [32]. The challenge is to find analgesics that reduce inflammatory pain and its associated hypersensitivity and, in the case of postoperative pain, prevent its chronicity but do not eliminate its warning or protective elements, a delicate and complex balance [3,6,32].

The use of knockout animals, evaluation of the kinin receptors’ protein expression, and studies of pathways underlying these receptors or crosstalk with other receptors could better confirm the role of kinins in the postoperative pain model. Advancement in this investigation is encouraged by the two previous clinical studies, which showed increased kinin levels and receptors expression in samples from oral surgery, suggesting that kinin receptors are involved in the early phase of inflammatory pain [17,18]. Hamza and colleagues [18] also showed an interaction between prostaglandins and kinins in this painful process and that up-regulation of the kinin B_1_ receptor may contribute to acute inflammatory pain through TRPV1 activation. Therefore, using kinin receptor antagonists associated with other drugs that target additional signaling pathways involved in pain and inflammation may be engaging in treating postoperative pain.

The multimodal approach is recommended for perioperative pain treatment, including various analgesics and techniques targeting different peripheral and central nervous system action mechanisms combined with nonpharmacological interventions [1,7]. In fact, neither pain nor recovery can be sufficiently treated by a single modality therapeutic due to the multifactorial characteristics of these processes [6]. Multimodal analgesic trials involving drug combinations acting at different receptors or techniques administrations have shown additive or synergistic effects, more effective pain relief, decreased opioid consumption, enhanced recovery, and reduced incidence of chronic postoperative pain compared with single-modality interventions [1,7,45]. Evidence supports the use of multimodal analgesia, although the exact components of its efficacy depend on the patient, setting, and surgical procedure [1].

Future analgesic studies based on the individuality of patients considering the preoperative nociceptive function, psychosocial risk factors, and specific procedures need to be prioritized [1,6,7]. For example, patients who come to the surgery with a pre-existing inflammatory condition may be at risk for an exaggerated postoperative inflammatory response and peripheral sensitization mediated by the kinin B_1_ receptor, which could initiate a chain of central sensitizing events that culminate in chronic pain [9]. Thus, in addition to considering studies that associate kinin receptors with other pharmacological targets to reduce postoperative pain and prevent its chronicity, it may be valid to analyze these effects in pre-existing pathologic conditions to surgical procedures.

Postoperative pain is a public health problem and a significant challenge due to morbidity and hospital expenses because previous treatment approaches have contributed substantially to the current opioid crisis [3,6,7]. So far, unrecognized, chronic postoperative pain has been included in the International Classification of Diseases—11th Revision (ICD-11), and this representation is expected to stimulate research that leads to improved treatments [33]. However, the pathophysiology of postoperative pain is unique, and the consequences are specific, which differs from other pain entities [7,19,45]. Thus, understanding the mechanisms underlying postoperative pain to identify more effective therapies with less risk of adverse effects may improve patients’ outcomes after surgery [7]. A more critical assessment of the scientific methods in the preclinical studies and randomized controlled trials is required to obtain better therapeutic strategies [6].

## 5. Conclusions

Although controversial, kinins seem to contribute mainly to the initial phase of inflammation and postoperative pain development. Thus, the kallikrein–kinin system may be a promising target to alleviate this pain. However, more investigations are needed, especially associations with other pharmacological targets considering the benefits of multimodal analgesia for postoperative pain.

## Figures and Tables

**Table 1:** Preclinical and clinical studies on the involvement of kinins and their receptors in postoperative pain.

**Preclinical studies**				
**Incision plantar model**	**Behavioural experiments**	**Treatments**	**Findings**	**Reference**
Longitudinal incision (1 cm) in the skin and fascia of the paw of rats, starting 0.5 cm from the proximal edge of the heel and extending toward the digits. The underlying muscle was dissected and submitted a single ongitudinal incision.	Mechanical hypersensitivity: circular plastic disk (5 mm in diameter) attached to a von electronic Frey filament (400 mN) applied three times in the paw. Five min after this nonpunctate testing, withdrawal responses to punctate mechanical stimulation were determined using von Frey filaments (15, 30, 54, 61, 94, 119, 142, 198, and 522 mN) applied the crescent manner until a withdrawal response or 522 mN (cut-off). Thermal hypersensitivity: withdrawal latencies to heat were assessed by applying a radiant heat source (50-W lamp).	Post-treatment with DALBk or HOE140 (0.1–3.0 mg/kg) intravenous via 2 h or 2 days after incision. Pre-treatment with DALBk or HOE140 (3.0 mg/kg) intravenous via 1 h before incision. One day after the surgical drugs were re-dosed.	None effect on mechanical or heat hypersensitivity evaluated from 0.5 to 2.5 h after treatments (post-treatment) or 2 h after incision or 1 h after re-dose (pre-treatment).	Leonard et al. (2004) [19].
Longitudinal incision (5 mm) in the skin and fascia of the paw mice and muscle was elevated and incised longitudinally.	Mechanical hypersensitivity: von Frey filaments were applied to the paw at an increasing force until the mouse withdrew its hind limb.	[des-Arg^10^]-HOE140 (0.2 mg/kg) and HOE140 (0.002–0.2 mg/kg) administered subcutaneously 30 min before incision. HOE140 (0.002—0.2 mg/kg) administered 5 min after the surgical procedure.	HOE140, but not [des-Arg^10^]-HOE140, reduced the mechanical hypersensitivity dose dependent from 2 h to 3 days after the operation (pretreatment). HOE140 (0.2 mg/kg), reduced mechanical hypersensitivity at 2 h after operation (post-treatment). None change kinin B_1_ and B_2_ receptors mRNA expression in the plantar tissue or spinal cord 24 h after the operation, or after pre-treatment with HOE140.	Muratani et al., 2005 [20].
The incision started 1 cm from the proximal edge of the heel of rats and extended longitudinally 1 cm toward the toes, intersecting the skin, fascia and plantar muscle.	Thermal hypersensitivity: noxious heat threshold temperature was determined in an increasing-temperature water bath initiating at 30 °C with a heating rate of 24 °C/min and cut-off of 53 °C.	[des-Arg^10^]-HOE140 and HOE140 (10 µM) intraplantar via 18 h after operation.	[des-Arg^10^]-HOE140 and HOE140 alleviated the heat hypersensitivity from 10 to 40 min after treatment.	Füredi et al., 2010 [21].
Longitudinal incision (5 mm) in the skin and fascia of the paw of mice and elevation of the underlying muscle. The incision started 2 mm from the proximal edge of the heel and extended toward the toes.	Mechanical hypersensitivity: manual von Frey filaments of increasing stiffness (0.02, 0.07, 0.16, 0.4, 1.4, 4.0 and 10.0 g). Six measurements were performed alternating the filaments conforming Oliveira et al., 2011 [27]; 2013 [28]; Brusco et al., 2017 [29].	DALBk (10–100 nmol/kg), Icatibant (HOE140; 300–1000 nmol/kg), SSR240612 (100–1000 nmol/kg) or FR173657 (30–300 nmol/kg), subcutaneous via 0.5 h before surgery DALBk (0.1–1 nmol/paw), Icatibant (HOE140; 1, 3, and 10 nmol/paw) or Aprotinin (100 µg/paw) intraplantar via 10 min before the surgery.	DALBk, Icatibant (HOE140), SSR240612 and FR173657 (systemic) or DALBk, Icatibant (HOE140) and Aprotinin (local) reduced the mechanical allodynia up to a maximum of 2 h after surgical. Bradykinin-related peptide levels increased in the serum and paw tissue.	Data of present study
**Clinical studies**				
**Findings**	**Reference**	**Findings**	**Reference**	** **
Increased kinin levels in the microdialysate samples obtained from after oral surgery in humans.	Swift et al., 1993 [17].	Increased kinin B_1_ and B_2_ receptor mRNA expression and kinin levels in mucosal biopsies three hours after oral surgery in humans.	Hamza et al., 2010 [18].	

## Data Availability

Not applicable.
